# Supplementary material for: Dataset from a human-in-the-loop approach to identify functionally important protein residues from literature
Source: Sci Data. 2024 Sep 27;11:1032. doi: 10.1038/s41597-024-03841-9 (PMC11436914; doi:10.1038/s41597-024-03841-9)
Supplement: Supplementary file 1 — Supplementary Information [file 41597_2024_3841_MOESM1_ESM.pdf]

Supplemental material to "Dataset from a human-in-the-loop approach to identify functionally important protein residues from literature"

Melanie Vollmar[1,*], Santosh Tirunagari[2], Deborah Harrus[1], David Armstrong[1], Romana Gaborova[3], Deepti Gupta[1], Marcelo Querino Lima Afonso[1], Genevieve Evans[1], and Sameer Velankar[1]

Affiliations

1. Protein Data Bank in Europe, European Molecular Biology Laboratory, European Bioinformatics Institute (EMBL-EBI), Wellcome Genome Campus, Hinxton, Cambridge, CB10 1SD, UK
2. Literature Services, European Molecular Biology Laboratory, European Bioinformatics Institute (EMBL-EBI), Wellcome Genome Campus, Hinxton, Cambridge, CB10 1SD, UK
3. CEITEC - Central European Institute of Technology, Masaryk University, Kamenice 5, 62500 Brno, Czech Republic

corresponding author(s): Melanie Vollmar (melaniev@ebi.ac.uk)

August 2024

batch	number of positive PubMed IDs	percent of total
1-10,000	8,682	87%
10,001-20,000	8,626	87%
20,001-30,000	8,612	86%
30,001-40,000	8,543	85%
40,001-50,000	8,601	86%
50,001-60,000	8,552	86%
60,001-70,000	8,543	85%
70,001-74,253	3,580	84%

Table S1. Proportion of publications selected from batches of 10,000 using seven independent LitSuggest models and a confidence score ≥ 0.8

Document ID	annotation count
PMC4784909[23]	865
PMC4786784[24]	1,549
PMC4792962[25]	1,268
PMC4832331[26]	739
PMC4833862[27]	1,044
PMC4848090[28]	987
PMC4850273[29]	1,121
PMC4850288[30]	716
PMC4852598[31]	1,229
PMC4887326[32]	933
Sum	10,451

Table S2. Total number of annotation counts for each of the initial ten manually annotated publications, after curation and consolidation. This is before converting the annotations into the IOB format for training.

Entity type	Total count	Unique mentions
Chemical	1,030	260
Complex Assembly	289	81
Evidence	645	155
Experimental Method	570	302
Gene	154	43
Mutant	614	188
Oligomeric State	151	23
Protein	1,457	122
Protein State	1,093	303
Protein Type	756	267
PTM	134	40
Residue Name	139	49
Residue Name Number	795	281
Residue Number	48	20
Residue Range	90	85
Site	519	209
Species	205	60
Structure Element	1,448	453
Taxonomy Domain	314	47
Sum	10,451	2,988

Table S3. Total and unique annotation count for the different entity types in the initial ten manually annotated publications, after curation and consolidation. This is before converting the annotations into the IOB format for training.

annotator	strict	exact	partial	type #	documents
annotator0	0.82	0.88	0.92	0.87	8
annotator1	0.70	0.80	0.87	0.74	2
annotator2	0.52	0.80	0.88	0.59	2
annotator3	0.55	0.65	0.79	0.69	2
annotator4	0.49	0.71	0.85	0.72	2
annotator5	0.49	0.86	0.92	0.53	2
annotator6	0.78	0.90	0.94	0.82	2

Table S4. Precision for manual annotation compared to ground truth for each annotator using SemEval evaluation

annotator	strict	exact	partial	type #	documents
annotator0	0.64	0.69	0.72	0.68	8
annotator1	0.39	0.44	0.49	0.41	2
annotator2	0.37	0.58	0.63	0.42	2
annotator3	0.43	0.50	0.62	0.61	2
annotator4	0.05	0.08	0.09	0.08	2
annotator5	0.14	0.24	0.25	0.14	2
annotator6	0.20	0.23	0.24	0.21	2

Table S5. Recall for manual annotation compared to ground truth for each annotator using SemEval evaluation

annotator	strict	exact	partial	type #	documents
annotator0	0.72	0.77	0.81	0.76	8
annotator1	0.50	0.57	0.62	0.53	2
annotator2	0.43	0.67	0.74	0.42	2
annotator3	0.48	0.57	0.69	0.61	2
annotator4	0.10	0.14	0.17	0.15	2
annotator5	0.21	0.37	0.40	0.23	2
annotator6	0.32	0.36	0.38	0.33	2

Table S6. F1-measure for manual annotation compared to ground truth for each annotator using SemEval evaluation

Table S7. Given are the PubMedCentral identifiers for the publications of the different data batches. An “x” indicates that a publication is included in the batch. The number of annotations for 19 and 20 entity types, where applicable, is also given. Given are the raw annotation counts after processing for model training.

Entity type	Batch 1 (19 entity types)	Batch 1 (20 entity types)	Batch 2 (19 entity types)	Batch 2 (20 entity types)	Batch 3 (20 entity types)	Batch 4 (20 entity types)	Batch 5 (20 entity types)
Bond Interaction	0	90	0	104	102	114	154
Chemical	1029	1030	1015	1025	1313	1229	1329
Complex Assembly	287	287	341	343	599	140	210
Evidence	639	642	804	804	1047	556	704
Experimental Method	569	569	718	718	659	537	826
Gene	153	153	47	47	1	8	44
Mutant	612	603	371	371	178	220	218
Oligomeric State	151	153	158	158	423	225	257
Protein	1446	1455	1681	1681	1590	1152	1686
Protein State	1092	1092	829	826	1107	944	803
Protein Type	753	753	479	481	537	566	720
PTM	134	141	31	73	53	145	79
Residue Name	139	139	122	122	113	242	85
Residue Name Number	795	792	566	524	610	423	451
Residue Number	48	48	30	30	68	8	5
Residue Range	90	90	69	69	102	63	93
Site	514	514	301	299	728	455	437
Species	203	203	106	106	288	178	155
Structure Element	1441	1441	1721	1721	1500	1574	1614
Taxonomy Domain	314	314	132	132	91	125	239
Total	10409	10509	9521	9634	11109	8904	10109

Table S8. Annotation counts for the different entity types in each batch after processing for model training.

Entity	v1.2	v1.4	v2.1	v3.1
Bond Interaction	-	-	**0.93**	0.82
Chemical	0.84	0.90	0.89	**0.92**
Complex Assembly	0.85	0.88	**0.91**	0.89
Evidence	0.74	0.86	0.84	**0.89**
Experimental Method	0.77	0.73	**0.85**	0.80
Gene	0.86	0.89	0.79	0.79
Mutant	0.83	0.93	0.91	**0.92**
Oligomeric State	0.94	0.88	0.93	**0.96**
Protein	0.91	0.97	0.94	**0.96**
Protein State	0.80	0.78	0.83	**0.86**
Protein Type	0.85	0.84	0.85	0.85
PTM	0.88	0.64	0.70	**0.85**
Residue Name	0.86	0.97	**0.92**	0.74
Residue Name Number	0.99	0.98	0.95	**0.96**
Residue Number	1.00	1.00	**0.80**	0.70
Residue Range	1.00	0.86	0.81	**0.89**
Site	0.83	0.83	0.85	**0.88**
Species	0.96	0.97	0.94	**0.95**
Structure Element	0.88	0.91	0.91	0.91
Taxonomy Domain	0.95	0.97	**0.99**	0.98

Table S9. Precision for the different models for the different entity types on the test set

Entity	v1.2	v1.4	v2.1	v3.1
Bond Interaction	-	-	0.88	**0.91**
Chemical	0.90	0.93	0.91	0.91
Complex Assembly	0.76	0.91	**0.93**	0.90
Evidence	0.76	0.89	0.88	0.88
Experimental Method	0.75	0.76	**0.85**	0.82
Gene	0.92	0.86	**0.86**	0.65
Mutant	0.92	0.95	**0.97**	0.94
Oligomeric State	1.00	1.00	0.99	**1.00**
Protein	0.93	0.97	**0.97**	0.96
Protein State	0.83	0.85	0.88	0.88
Protein Type	0.84	0.90	0.85	**0.88**
PTM	0.76	0.81	0.70	**0.79**
Residue Name	0.95	0.92	**0.97**	0.96
Residue Name Number	0.99	0.99	0.96	**0.98**
Residue Number	1.00	0.93	**0.97**	0.73
Residue Range	0.80	0.91	0.70	**0.86**
Site	0.82	0.86	0.87	**0.90**
Species	0.98	1.00	**0.96**	0.95
Structure Element	0.86	0.92	0.92	0.92
Taxonomy Domain	0.97	0.96	0.98	0.98

Table S10. Recall for the different models for the different entity types on the test set

Entity	v1.2	v1.4	v2.1	v3.1
Bond Interaction	-	-	**0.90**	0.86
Chemical	0.87	0.92	0.90	**0.92**
Complex Assembly	0.80	0.89	**0.92**	0.90
Evidence	0.75	0.88	0.86	**0.89**
Experimental Method	0.76	0.75	**0.85**	0.81
Gene	0.89	0.88	**0.82**	0.71
Mutant	0.88	0.94	**0.94**	0.93
Oligomeric State	0.97	0.93	0.96	**0.98**
Protein	0.92	0.97	0.95	**0.96**
Protein State	0.81	0.81	0.85	**0.87**
Protein Type	0.84	0.87	0.85	**0.87**
PTM	0.81	0.71	0.70	**0.82**
Residue Name	0.91	0.94	**0.94**	0.84
Residue Name Number	0.99	0.99	0.96	**0.97**
Residue Number	1.00	0.96	**0.88**	0.71
Residue Range	0.89	0.89	0.75	**0.87**
Site	0.82	0.85	0.86	**0.89**
Species	0.97	0.98	0.95	0.95
Structure Element	0.87	0.91	**0.92**	0.91
Taxonomy Domain	0.96	0.97	0.98	0.98

Table S11. F1-measure for the different models for the different entity types on the test set

model	data batch	strict	exact	partial	type
v1.2	batch 2	0.66	0.75	0.83	0.75
v1.4	batch 3	0.69	0.79	0.85	0.75
v2.1	batch 4	0.75	0.83	0.88	0.82
v2.1	batch 5	0.78	0.85	0.91	0.85
v3.1	batch 5	0.77	0.84	0.90	0.86

Table S12. Precision for models and their respective publication batches compared to ground truth for each batch using SemEval evaluation

model	data batch	strict	exact	partial	type
v1.2	batch 2	0.67	0.77	0.85	0.76
v1.4	batch 3	0.71	0.80	0.87	0.77
v2.1	batch 4	0.75	0.83	0.89	0.83
v2.1	batch 5	0.75	0.82	0.87	0.81
v3.1	batch 5	0.75	0.79	0.86	0.81

Table S13. Recall for models and their respective publication batches compared to ground truth for each batch using SemEval evaluation

model	data batch	strict	exact	partial	type
v1.2	batch 2	0.67	0.76	0.84	0.76
v1.4	batch 3	0.70	0.79	0.86	0.76
v2.1	batch 4	0.75	0.83	0.89	0.82
v2.1	batch 5	0.76	0.83	0.89	0.83
v3.1	batch 5	0.75	0.81	0.88	0.83

Table S14. F1-measure for models and their respective publication batches compared to ground truth for each batch using SemEval evaluation
